# Improving Stroke Outcome Prediction Using Molecular and Machine Learning Approaches in Large Vessel Occlusion

**DOI:** 10.3390/jcm13195917

**Published:** 2024-10-03

**Authors:** Madhusmita Rout, April Vaughan, Evgeny V. Sidorov, Dharambir K. Sanghera

**Affiliations:** 1 Department of Pediatrics, College of Medicine, University of Oklahoma Health Sciences Center, Oklahoma City, OK 73104, USA; madhusmita-rout@ouhsc.edu; 2 Department of Neurology, University of Oklahoma Health Sciences Center, Oklahoma City, OK 73104, USA; april-vaughan@ouhsc.edu; 3 Department of Pharmaceutical Sciences, University of Oklahoma Health Sciences Center, Oklahoma City, OK 73104, USA; 4 Department of Physiology, College of Medicine, University of Oklahoma Health Sciences Center, Oklahoma City, OK 73104, USA; 5 Oklahoma Center for Neuroscience, University of Oklahoma Health Sciences Center, Oklahoma City, OK 73104, USA; 6 Harold Hamm Diabetes Center, University of Oklahoma Health Sciences Center, Oklahoma City, OK 73104, USA

**Keywords:** large vessel occlusion, outcome, infarct volume, microRNAs, metabolites, machine learning

## Abstract

**Introduction**: Predicting stroke outcomes in acute ischemic stroke (AIS) can be challenging, especially for patients with large vessel occlusion (LVO). Available tools such as infarct volume and the National Institute of Health Stroke Scale (NIHSS) have shown limited accuracy in predicting outcomes for this specific patient population. The present study aimed to confirm whether sudden metabolic changes due to blood-brain barrier (BBB) disruption during LVO reflect differences in circulating metabolites and RNA between small and large core strokes. The second objective was to evaluate whether integrating molecular markers with existing neurological and imaging tools can enhance outcome predictions in LVO strokes. **Methods**: The infarction volume in patients was measured using magnetic resonance diffusion-weighted images, and the 90-day stroke outcome was defined by a modified Rankin Scale (mRS). Differential expression patterns of miRNAs were identified by RNA sequencing of serum-driven exosomes. Nuclear magnetic resonance (NMR) spectroscopy was used to identify metabolites associated with AIS with small and large infarctions. **Results**: We identified 41 miRNAs and 11 metabolites to be significantly associated with infarct volume in a multivariate regression analysis after adjusting for the confounders. Eight miRNAs and ketone bodies correlated significantly with infarct volume, NIHSS (severity), and mRS (outcome). Through integrative analysis of clinical, radiological, and omics data using machine learning, our study identified 11 top features for predicting stroke outcomes with an accuracy of 0.81 and AUC of 0.91. **Conclusions**: Our study provides a future framework for advancing stroke therapeutics by incorporating molecular markers into the existing neurological and imaging tools to improve predictive efficacy and enhance patient outcomes.

## 1. Introduction

Stroke is the third-leading cause of death and disability worldwide, and acute ischemic stroke (AIS) accounts for 85% of all strokes. According to estimates from the Global Burden of Diseases, Injuries, and Risk Factors Study, AIS mortality led to approximately 6.55 million deaths worldwide from 1990 to 2019 [1]. Large vessel occlusions (LVOs) in the AIS occur due to sudden interruption of blood flow in the large artery due to atherothrombosis or cardiac embolism, which leads to rapid death of neurons and neurological deterioration [2,3,4]. LVOs are identified based on occlusion in the internal carotid artery, the first and second segments of the middle cerebral artery, the basilar artery, the vertebral artery, the first and second segments of the posterior cerebral artery, and the first segment of the anterior cerebral artery [5]. Patients with LVOs constitute a significant subpopulation of AIS, and their annual incidence in the United States is around 80,000 [6].

At present, stroke is diagnosed based on clinical assessments and radiological exams by magnetic resonance imaging (MRI) and computed tomography (CT), along with currently available treatment therapies, including reperfusion and endovascular thrombectomy (EVT). The severity of a stroke is typically assessed using the National Institute of Health Stroke Scale (NIHSS) scores, while the outcome is evaluated with the modified Rankin Scale (mRS) [7]. The infarct volume is an important determinant of long-term consequences in the AIS, leading to extensive neuronal damage and subsequent neuronal deficit. Even though the infarct volume strongly correlates with clinical outcomes, it cannot explain why some patients after large infarctions demonstrate significant improvement while others develop poor outcomes with small strokes. There is currently limited understanding of the connection between infarct volume, severity, and outcomes [8]. 

Recent improvements in high-throughput metabolomic technologies (liquid-chromatography and mass spectroscopy (LCMS) and nuclear magnetic resonance (NMR)) and transcriptomics have made it possible to characterize molecular changes in multiple tissues of a large number of patients at the same time. Noncoding regions of the genome encoding RNA, microRNAs (miRNAs), play an important role in regulating the metabolic homeostasis of different diseases by influencing the regulation of target genes [9]. MiRNAs have been implicated in the disruption of the blood–brain barrier (BBB) and induce cellular changes in AIS, including mitochondrial dysfunction, cytokine-mediated cytotoxicity, oxidative stress, glial cell activation, and inflammation [10]. Thus far, the majority of biomarker discoveries in AIS have mainly been carried out in rodents utilizing middle cerebral artery occlusion (MCAO) models. However, their predictability in human strokes has proven to be challenging [11,12]. There is a need to identify chemical/biological markers to improve stroke prognostics and accurately predict the stroke outcomes following EVT or thrombolysis. During the acute phase of the LVO, the aberrant expression of the extracellular transcriptome and alterations in metabolite species in the brain regulate neuroinflammation, causing BBB interruption. The present study aimed to confirm whether sudden metabolic changes due to BBB disruption reflect differences in circulating metabolites and RNA between small and large core strokes. The second objective was to utilize machine learning as a predictive tool to integrate clinical, radiological imaging, transcriptomic (miRNA), and metabolomic data to determine functional outcomes in patients with LVO strokes.

## 2. Experimental Procedure

### 2.1. Study Participants 

**Figure 1:**
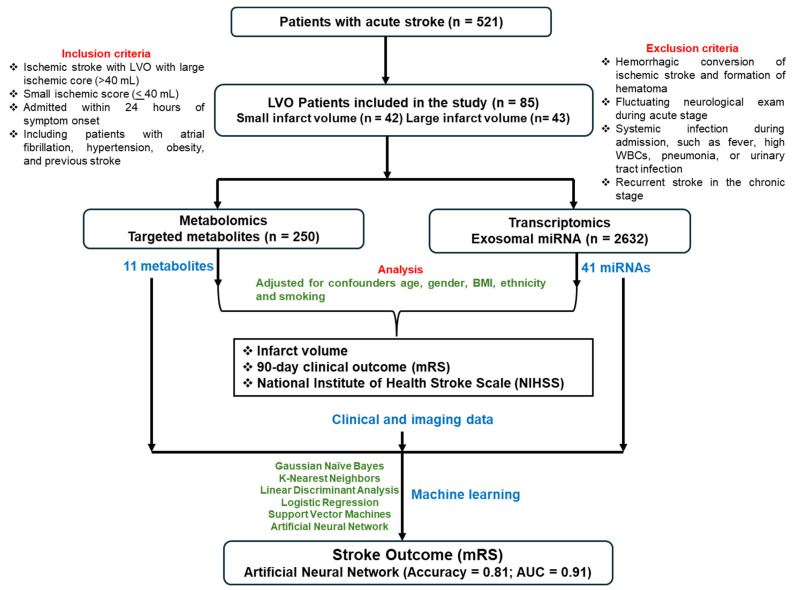
Flow chart of the study.

The study comprised 85 LVO patients who were part of our ongoing Metabolome in Ischemic Stroke Study (MISS), which began in 2017 for recruiting patients from the Comprehensive Stroke Center at the Oklahoma University Medical Center. The MISS is designed to identify biomarker predictors of AIS and other strokes using metabolomics and other omics technologies in longitudinal and cross-sectional study cohorts [13,14,15,16]. The blood samples were collected during acute (<72 h of stroke) and follow-up phases (within 3 months). All patients used for this cross-sectional study underwent MRI with diffusion-weighted imaging (DWI) sequence during admission for AIS definition. Hemorrhagic stroke was classified based on subarachnoid and intracerebral hemorrhage. Patients were evaluated upon admission with the NIHSS (0–42 points reflecting the least to the most severe neurological deficit) and then with the Miami Emergency Neurological Deficit examination to monitor for signs of neurological deterioration during hospital admission at the acute stage [17,18]. The inclusion criteria comprised (i) ischemic strokes with LVO with a large ischemic core (>40 mL), (ii) a small ischemic core (<40 mL) admitted within 24 h of symptom onset, and (iii) patients with atrial fibrillation, hypertension, obesity, and previous stroke. We excluded those patients with (i) hemorrhagic conversion of ischemic stroke and formation of hematoma defined by the European Cooperative Acute Stroke Study [19], (ii) fluctuating neurological exams during the acute stage, (iii) systemic infection at presentation or during admission with fever >38.0 °C, elevated white blood cells, diagnosis of pneumonia, or urinary tract infection, (iv) renal disease with a glomerular filtration rate (GFR) <45; and (v) recurrent stroke in the chronic stage that reduces confounding due to these conditions as described [15]. Of the 85 LVO cases, 14% had atrial fibrillation, 64% had hypertension, 40% were obese (BMI > 30), 45% were diabetic, 19% had coronary artery disease (CAD), and 24% had a previous history of AIS. The University of Oklahoma Health Sciences Center’s Institutional Review Board approved all study protocols. Figure 1 depicts the workflow of the study.

### 2.2. Neuroimaging, Infarct Volume and Clinical Outcome 

Two independent investigators measured stroke (ischemic core) volume using MR diffusion-weighted images 12 to 48 h after admission using MR segmentation software [15]. As described in our earlier study, this software utilizes an interactive computer-aided detection (CAD) scheme. The user identifies and draws the stroke boundary on each of the identified stroke slices, and the CAD scheme computes the resulting infarction volume [15]. The infarct volume cut-off was used to categorize small (≤40; N = 42) and large (>40; N = 43) infarct volumes. The mRS assesses the clinical outcome of AIS patients after the stroke [20,21]. It grades the patient’s disability from 0 (no symptoms) to 6 (death), thus capturing the whole spectrum of functional states 30 and 90 days following stroke [20,22,23]. 

### 2.3. Metabolome Analysis 

Aliquots of frozen serum from MISS patients were shipped on dry ice to Nightingale Health Ltd., Helsinki, Finland (https://nightingalehealth.com/), to measure lipidomics profiling using targeted high-throughput NMR. This platform provided simultaneous quantification of 250 metabolic measures, routine lipids, lipoprotein subclass profiling with lipid concentrations within 14 subclasses, esterified fatty acid composition, and various low molecular metabolites, including amino acids, ketone bodies, and gluconeogenesis-related metabolites in molar concentration units as described previously [24,25]. 

### 2.4. Exosome Extraction and RNA Isolation

The blood sample collected in anti-coagulant-free tubes was centrifuged for 10 min at 1258× *g* (4 °C), and the supernatant (serum) was transferred into a fresh tube and stored at –80 °C until further analysis. Exosomal miRNA quantification and differential expression analysis were performed using serum specimens. Following the manufacturer’s protocol, the Serum/Plasma Exosome Kit (Qiagen Inc., Chatsworth, CA, USA) was used. We first characterized the particle size morphology of the exosomes extracted from the sera of patients using transmission electron microscopy and via nanoparticle tracking analysis (NTA) using Nanosight 300 as described earlier [26]. Further, the exosome pellet was resuspended in a resuspension buffer to extract the total RNA. RNA was extracted and purified using the miRNeasy mini kit (Qiagen Inc., Chatsworth, CA, USA) and ExoQuick Exosome Isolation and RNA Purification Kit (System Biosciences, Palo Alto, CA, USA) according to the manufacturer’s protocol.

### 2.5. miRNA Sequencing Quality Control (QC) and Analysis

RNA concentrations were estimated and validated for their quantity and integrity using Agilent’s autoanalyzer. RNA quality was determined using a tape station. The nextGen sequencing libraries were created using QIAGEN’s QIAseq miRNA library kit, which incorporates Unique Molecular Indices, and the QC of libraries was performed using Agilent’s bioanalyzer to determine library size and quality. Sequencing was performed on an Illumina sequencer using single-end, 75 bp reads to a depth of 12–14 million reads at the Genomics Core facility of the Oklahoma Medical Research Foundation (OMRF). Sequence reads were pre-processed to remove adapters and were aligned to the reference human genome (hg19) using the Bowtie aligner as described [27,28]. The reads aligning to each known mature miRNA were counted using Bioconductor packages for next-generation sequencing data analysis [29] based on miRNA definitions in the miRBase database [30]. Sequencing analysis was performed using QIAGEN’s CLC workbench.

### 2.6. Statistical Analysis 

The clinical and demographic characteristics of the study participants were analyzed using the phenotypic mean for continuous variables and percentages for categorical variables. We characterized and prioritized differentially expressed miRNAs correlating with the acute stroke using the normalized reads and a regression approach (R package DESeq2). A series of standardization steps were performed to make the expression phenotypes comparable across individuals and across miRNAs. To minimize the influence of overall signal levels, abundance values of all retained miRNAs were standardized within individual z-scores, followed by linear regression against individual-specific average log-transformed raw signals and their squared value. Before the statistical analysis, the raw miRNA and metabolite profile data were normalized through log transformation, which resulted in some negative values and was scaled by MetaboAnalyst 5.0 (www.metaboanalyst.ca/). We first used two-tailed, unpaired Wilcoxon rank-sum tests with *p* < 0.05 to identify metabolites and miRNAs that differed significantly in small and large infarct volumes using MetaboAnalyst. A false discovery rate (FDR) was implemented to correct for the multiple comparisons. Multiple linear regression analyses were performed to assess the impact of individual miRNA and metabolite markers on infarct volume after adjusting for covariates such as age, gender, ethnicity, body mass index (BMI), smoking, etc. Pearson correlation analysis was performed to determine the linear association between miRNAs and metabolites and the association of mRS and NIHSS scores with miRNAs and metabolites. All analyses were performed using SVS version 8.9.1 (Golden Helix, Bozeman, MT, USA) and SPSS version 29. For a sample size of 43 patients with large-volume strokes and 42 patients with small-volume strokes, with a significance level (alpha) of 2.0 × 10^−4^ and 250 metabolites, our study has 80% power to detect a mean difference of 0.58 standard deviation (SD) between small- and large-volume groups. After assuming conservative multi-testing correction for ~2600 miRNA transcripts at a significant level of 1.92 × 10^−5^ for the same patients, our study will achieve ~75% power to detect a mean difference of 1 SD to identify miRNAs differing between groups using a two-sided two-sample equal-variance *t*-test.

### 2.7. Machine Learning

Machine learning was used as a predictive tool to integrate clinical, miRNAs, and metabolomics biomarker data differing among 68 stroke cases in good (mRS = 0–3) and bad (mRS = 3–6) stroke outcomes. A binary classification method was employed to determine stroke outcomes in Python (v3.11.5) using sci-kit-learn [31] and pandas libraries. In binary classification, class labels were determined by stroke cases with mRS (≤3; N = 37) characterized as a good outcome and those with mRS (>3; N = 31) characterized as a bad outcome. Chi-Square and F-regression tests were used for selecting features, and the Shapley Additive exPlanations (SHAP) framework from Slundberg (https://github.com/slundberg/shap accessed on 21 June 2024) was used to identify important features using an XGBoost model with a Python 3 kernel (v3.11.5). Summary plots depict the top influential biomarkers and how they influence the model prediction [32,33]. Among the 216 miRNAs and 250 metabolites, only those features that were highly dependent on the outcome were selected. The 11 selected features were scaled as initial QC to improve the performance and accuracy of machine learning algorithms. Scaling ensures all features contribute equally by converting them to a common scale to eliminate potential biases. The data file was split into 80% training and 20% testing partitions using a defined seed value. Seeds were only chosen such that the resulting training and testing accuracies were similar, preventing overfit to the small sample size. Tenfold cross-validation was used to evaluate the model and detect overfitting. Supervised learning classifiers such as Logistic Regression (LR), Linear Discriminant Analysis (LDA), K-Nearest Neighbors Classifier (KNN), Gaussian Naïve Bayes (NB), Support Vector Machine (SVM) and a deep learning method, Artificial Neural Network (ANN), were used to classify the stroke outcome. The model was evaluated using various classification metrics, such as accuracy, which is defined as the number of correct predictions divided by the total number of predictions. The receiver operator characteristics (ROC) curve is a measure of sensitivity and specificity at different decision thresholds, and the area under the curve (AUC) offers a single metric for summarizing the overall performance of the model. 

## 3. Results

**Figure 2:**
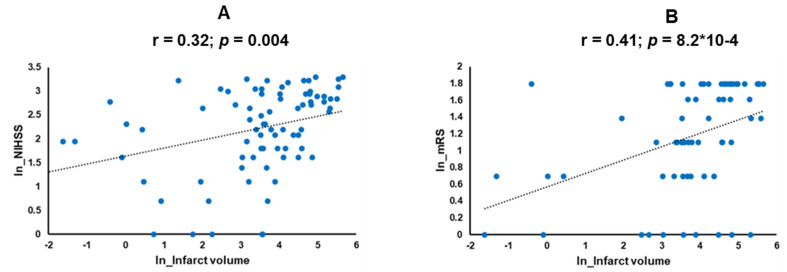
Correlation analysis in the scatter plots shows correlations between (**A**) NIHSS with infarct volume and (**B**) mRS with infarct volume in the log-transformed data values of NIHSS and mRS. NIHSS: National Institute of Health Stroke Scale; mRS: modified Rankin Score.

The clinical and demographic characteristics of the study participants are presented in Table 1. There were no significant differences in the average age, BMI, and diastolic blood pressure between large and small infarct volume cases. Plasma glucose was significantly elevated (162.9 ± 96.7) in cases with large infarcts compared to small infarcts (128.5 ± 59.3). Systolic blood pressure was significantly higher in large (158.1 ± 32.7) vs. small strokes (145.7 ± 21.9). The study included predominantly Caucasian participants (93% and 74%), with smaller percentages of African Americans (7% and 14%) in the large and small infarct volume groups, respectively. The prevalence of type 2 diabetes was 53% in individuals with large infarct volumes and 36% in those with small infarct volumes. Smoking was prevalent in patients with small infarcts (50%) compared to those with large infarcts (44%). The average infarct volume differed significantly between large (117.4 ± 69.1) and small (19.1 ± 14.1) strokes (*p* = 7.43 × 10^−12^). Notably, patients with a large infarct volume had significantly worse outcomes, as indicated by the average mRS of (4.2 ± 1.9) compared to the mRS of (2.6 ± 1.9) of those with a small infarct volume (*p* = 0.0004) (Table 1). Incidentally, our data revealed a stronger correlation between the infarct volume and mRS (r = 0.41; *p* = 8.2 × 10^−4^) than the infarct volume and NIHSS (r = 0.32; *p* = 0.004) (Figure 2). 

### 3.1. Discovery of Significantly Altered Patterns of Circulating miRNAs Associated with Infarct Volume 

**Figure 3:**
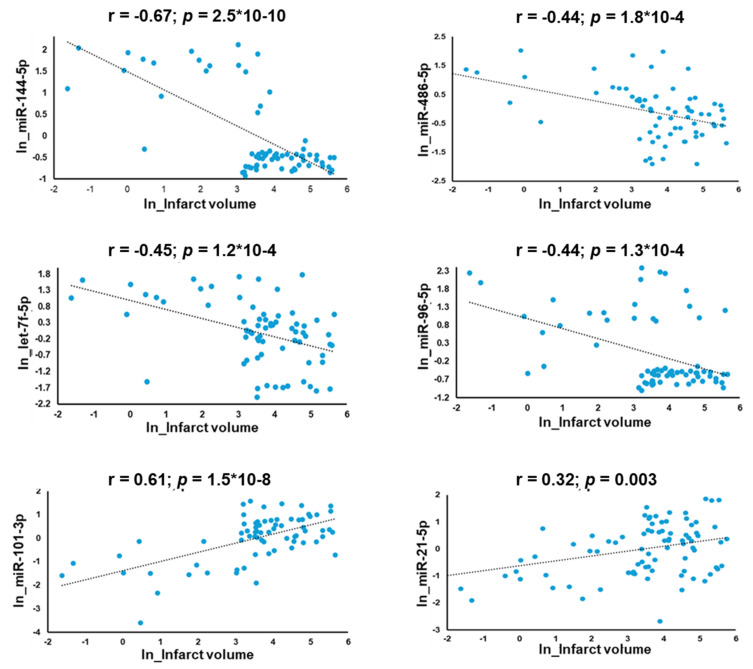
Scatter plots of correlation analysis depicting the miRNAs (miR-144-5p, miR-486-5p, miR-96-5p and let-7f-5p) whose expression was downregulated in patients with large infarcts and miRNAs (miR-101-3p and miR-21-5p), which were upregulated with increased infarct volume.

Sequencing analysis of exosomal miRNAs identified 2632 total miRNAs in our samples. Of these, 41 miRNAs were differentially expressed between large and small infarct volumes. Most of the miRNAs were significantly negatively regulated in large infarcts except for a few, which were upregulated in large infarcts: miR-21-5p (t.stat = 3.5; *p* = 7.1 × 10^−4^), miR-101-3p (t.stat = 3.8; *p* = 2.5 × 10^−4^), and miR-16-5p (t.stat = 2.3; *p* = 0.03) (Supplementary Table S1; Figure 3). Selected miRNAs such as miR-144-5p (r = −0.67; *p* = 2.5 × 10^−10^), miR-486-5p (r = −0.44; *p* = 1.8 × 10^−4^), let-7f-5p (r = −0.45; *p* = 1.2 × 10^−4^) and miR-96-5p (r = −0.44; *p* = 1.3 × 10^−4^) showed a strong negative correlation with infarct volume (Figure 3). 

### 3.2. Association of miRNAs with NIHSS and Stroke Outcome (mRS Score)

The miRNAs that were significantly associated with high infarct volume also correlated with NIHSS and stroke outcomes (mRS). The majority of the miRNAs showed a significant negative correlation with NIHSS scores. The top ones were miR-625-3p (r = −0.51; *p* = 9.8 × 10^−7^) and miR-486-5p (r = −0.41; *p* = 1.8 × 10^−4^). However, miR-16-5p (r = 0.52; *p* = 8.5 × 10^−7^) displayed a positive correlation with NIHSS (Supplementary Table S2). Additionally, eight miRNAs were negatively correlated with increased mRS (Supplementary Table S3). 

### 3.3. Discovery of Significantly Altered Patterns of Serum Metabolites with Infarct Volume 

Selected metabolites such as glucose (r = 0.42; *p* = 3.6 × 10^−4^) and acetoacetate (r = 0.33; *p* = 0.006) were positively associated with infarct volume (Supplementary Figure S1). Multivariate regression analysis for infarct volume identified 11 metabolites, mostly lipids, to be associated with stroke volume (Table 2). Glucose (Beta (SE): 0.51 (0.17); *p* = 3.2 × 10^−3^), free cholesterol to total lipids ratio in small HDL (S-HDL-FC%) (Beta (SE): 0.53 (0.18); *p* = 4.6 × 10^−3^) and ketone bodies such as acetoacetate (Beta (SE): 0.36 (0.16); *p* = 0.03) were increased in patients with a high stroke volume (Table 2). 

### 3.4. Association of Metabolites with NIHSS and Stroke Outcomes (mRS)

Since NIHSS correlated with the infarction volume in this study, it was not surprising to find metabolites associated with the infarct volume also correlated with NIHSS. Metabolites of ketone bodies such as acetoacetate (r = 0.35; *p* = 5.2 × 10^−3^) and lipid S-HDL-FC% (r = 0.29; *p* = 0.02) showed a positive significant correlation with NIHSS, while glycerol (r = −0.31; *p* = 0.01) and albumin (r = −0.27; *p* = 0.03) correlated negatively with NIHSS (Supplementary Table S4). Interestingly, ketone bodies acetoacetate (r = 0.43; *p* = 1.2 × 10^−3^) and 3-hydroxybutyrate (r = 0.34; *p* = 0.01) were positively correlated with mRS, depicting poor clinical outcomes (Supplementary Table S5).

### 3.5. Association of Exosomal miRNAs with Metabolites 

**Figure 4:**
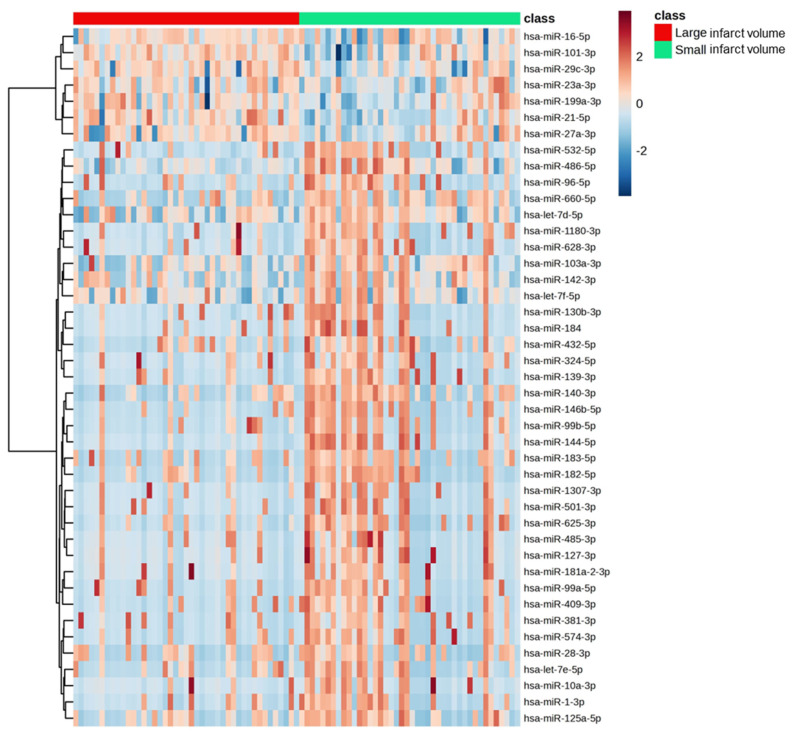
Heat maps depicting the differential regulation of significant miRNAs in large and small infarct volumes.

We identified 41 miRNAs that were significantly differentially regulated with respect to infarct volume with exceptions for miR-21-5p, miR-27a-3p, miR-29c-3p, miR-101-3p, and miR-16-5p (Figure 3 and Figure 4). In total, 21 metabolites showed a significant correlation with 41 miRNAs. Amino acids such as alanine (Ala), glycine (Gly), and histidine (His) correlated positively with miRNAs, whereas glycoprotein acetyls (GlycA), glutamine (Gln), and phenylalanine (Phe) were negatively correlated with the miRNAs that were down-regulated in large infarct strokes. Interestingly, the upregulated miRNAs (miR-101-3p, miR-16-5p, and miR-21-5p) in large infarct strokes correlated positively with ketone bodies, glucose, apolipoprotein B (ApoB) and phospholipids in small LDL (S-LDL-PL) (Supplementary Figure S2).

**Figure 5:**
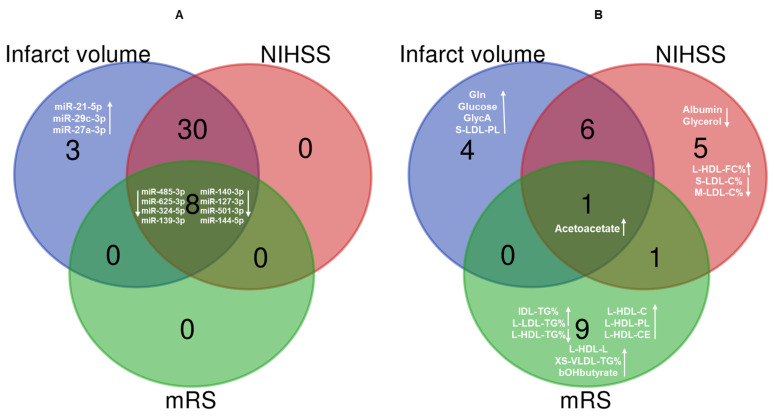
Venn diagrams showing the most relevant (**A**) miRNAs and (**B**) metabolites associated with infarct volume, NIHSS, and mRS. NIHSS: National Institute of Health Stroke Scale; mRS: modified Rankin Score; Gln: glutamine; GlycA: glycoprotein acetyls; S-LDL-PL: phospholipids in small LDL; L-HDL-FC%: free cholesterol to total lipids ratio in large HDL; S-LDL-C%: cholesterol to total lipids ratio in small LDL; M-LDL-C%: cholesterol to total lipids ratio in medium LDL; IDL-TG%: triglycerides to total lipids ratio in IDL; L-LDL-TG%: triglycerides to total lipids ratio in large LDL; L-HDL-TG%: triglycerides to total lipids ratio in large HDL; L-HDL-C: cholesterol in large HDL; L-HDL-PL: phospholipids in large HDL; L-HDL-CE: cholesteryl esters in large HDL; L-HDL-L: total lipids in large HDL: XS-VLDL-TG%: triglycerides to total lipids ratio in very small VLDL; bOHbutyrate: 3-Hydroxybutyrate. Upward arrow- increased metabolite level/miRNA expression; Downward arrow- decreased metabolite level or miRNA expression.

Overall, eight miRNAs (miR-485-3p, miR-625-3p, miR-324-5p, miR-139-3p, miR-140-3p, miR-127-3p, miR-501-3p and miR-144-5p) and one metabolite (acetoacetate) correlated strongly with infarct volume, NIHSS, and mRS (Figure 5). The increased expression of miR-21-5p, miR-29c-3p, and miR-27a-3p and increased levels of Gln, GlycA, glucose, and S-LDL-PL correlated only with infarct volume (Figure 5).

### 3.6. Machine Learning Analysis

**Figure 6:**
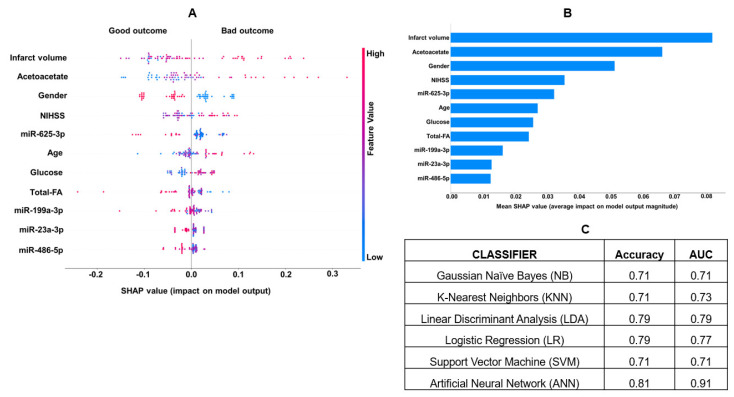
(**A**) The most important predictive parameters using binary classification with mRS, with the absolute value of a feature being high (red) or low (blue), depicting bad or good stroke outcomes. (**B**) The VIP plot shows the most influential biomarkers and their impact on stroke outcomes. (**C**) Machine learning analysis for clinical, miRNAs, and metabolite features using binary classification and artificial neural networks was the most informative model showing high sensitivity and specificity in AUC. NIHSS: National Institute of Health Stroke Scale; FA: fatty acids; mRS: modified Rankin Scale.

The clinical, radiological, transcriptomic, and metabolic markers that showed the strongest association were utilized in the final analyses for predicting stroke outcomes. Feature selection using statistical tests such as Chi-square or F-regression tests identified nine metabolites and seven miRNAs that could be used as features for predicting stroke outcomes (Supplementary Table S6). Upon further analysis using SHAP, we identified 11 interactive features with a higher impact on the model. These included clinical parameters such as age, gender, infarct volume, and NIHSS, miRNAs including miR-625-3p, miR-23a-3p, miR-486-5p, and miR-199a-3p, and metabolites like acetoacetate, glucose, and total fatty acids (total-FA) as detailed in Figure 6A,B. The combined list of features was used in tenfold cross-validation. The ANN was the most powerful classifier, with an accuracy of 0.81 and an AUC of 0.91. It was followed by LDA with an accuracy of 0.79 and an AUC of 0.79, LR with an accuracy of 0.79 and an AUC of 0.77, and KNN with an accuracy of 0.71 and an AUC of 0.73. NB and SVM had similar accuracies and AUCs, both with an accuracy of 0.71 and an AUC of 0.71 for predicting stroke outcomes (Figure 6C).

## 4. Discussion

The disruption of the BBB leads to rapid changes in circulatory fluids. We hypothesize that these changes in metabolites and small extracellular RNAs in the bloodstream are associated with the size of the stroke. Measuring these changes might provide additional predictive value when combined with clinical and imaging tools to determine clinical outcomes. Our study showed that the infarct volume was a stronger determinant of stroke outcomes (r = 0.41; *p* = 8.2 × 10^−4^) than the NIHSS (r = 0.32; *p* = 0.004) (Figure 2). A strong correlation between NIHSS and infarct volume (r = 0.82; *p* < 0.0001) reported by Furlanis et al. [34] earlier could not be confirmed in our study (r = 0.32; *p* = 0.004). NIHSS and volume have been shown to predict stroke outcomes in AIS patients treated with intravenous thrombolysis [35]. Intravenous (IV) tissue plasminogen activator (rt-PA), a clot-dissolving drug, is effective in treating AIS and improving survival outcomes; however, the use of IVtPA for the majority of cases remained restricted to 4.5 h after the onset [21]. A recent study by Popa et al. showed that the door-to-CT time was the only factor significantly correlated with the death outcomes in comparison to door-to-physician time, NIHSS at admission, or after 24 h [36]. EVT is a mechanical procedure to remove a thrombus from the blocked brain artery during the AIS. In 2017, the use of EVT can be extended to 24 h in selected patients with small ischemic cores (<40mL). However, in patients with large ischemic cores, the application of EVT procedures showed higher rates of vascular and hemorrhagic complications leading to disabilities in recent clinical trials [37,38,39]. Infarct volume has been shown to discriminate patient outcomes in mild strokes in earlier studies [40], but no data are available on large core strokes. Therefore, given the limitations of existing technologies and stringency in the therapeutic window, the discovery of actionable molecular markers and their integration into the existing radiological and clinical protocols can improve the accurate prognostication of outcomes in patients with large ischemic cores. 

Of the 41 identified small RNAs in patients with large infarct volumes, miR-144-5p was the most downregulated considerably (log_2_FC = −4.55; *p* = 1.1 × 10^−5^) in large strokes, and miR 144-5p and miR-625-3p showed a significant negative association with clinical outcome (mRS). Studies on animal models have shown miR-144 to have a protective role against neuroinflammation and oxidative stress by targeting the pathway of miR-451-14-3-3ζ-FoxO3 in intracerebral hemorrhage. [41]. Additionally, miR-144 downregulates PTEN and upregulates Akt in mice, supporting its protective role in AIS [42]. Decreased miR-625 expression was observed in laryngeal squamous cell carcinoma tissues, breast cancer, and melanoma, but some studies reported conflicting results [43,44,45,46]. Elevated serum levels of miR-21 were attributed to a 6.2-fold increase in stroke risk in patients with AIS [47]. MiR-21 was increased in patients with subarachnoid hemorrhage [48] and cerebral ischemia, leading to increased ERK-stimulated MMP9 levels [49]. 

Several circulating metabolites were differentially regulated with respect to infarct volume in AIS. Patients with large infarct sizes showed significantly increased levels of glucose and ketone bodies. Hyperglycemia and ketone bodies were highly prevalent in AIS in our patients from Oklahoma and the UK biobank [16,25]. In this study, glucose and ketone bodies were associated with poor outcomes supported by other published studies [50,51,52]. Urine ketone bodies were also positively correlated with recurrent stroke [53]. Multivariate regression and correlation analysis identified that eight miRNAs and one metabolite correlated with all clinical and neurological factors, including infarct volume, stroke severity (NIHSS), and outcomes (Figure 5). Through integrative analysis using machine learning, our study identified and confirmed the top 11 features to predict stroke outcomes by machine learning (Figure 6A,B). ANN was the most powerful classifier for predicting poor (mRS > 3) and good (mRS = 0 to 3) outcomes with an accuracy of (0.81) and AUC of (0.91). Notably, miR-199a-3p and miR-23a-3p identified by machine learning were not among the top miRNAs found in statistical analysis. MiR-199a-5p promotes functional recovery after cerebral ischemia by inhibiting Cav-1 to facilitate neurogenesis; it has been shown to be downregulated in rats with cerebral ischemia [54,55]. Similarly, miR-23a-3p lowers oxidative stress in cerebral ischemia [56]. Fatty acids, such as omega-6, mono- and poly-unsaturated fatty acids, and saturated fatty acids, were significantly decreased in AIS and inversely correlated with the infarct volume, as we reported earlier [25].

Besides molecular, clinical, and radiological factors, our interactive machine learning models identified gender to be a significant determinant of stroke outcomes. Male patients with small and large cores exhibited good outcomes compared to women (Figure 6). Sex differences have been shown to determine the treatment and outcomes of AIS [57]. Even though women had better survival rates after AIS, they also were more disabled and experienced poor quality of life in a study reported on 19000 patients [58]. Increased age was also a strong predictor of poor outcomes in our study (Figure 6), which is an important unmodifiable factor for poor recovery and neurological worsening in AIS. Age-related risk is modulated by gender and other risk factors in our study, as supported by earlier studies [59]. Machine learning and deep learning algorithms can identify outliers within the small patient datasets that may influence disease diagnosis [60,61]. To avoid overfitting, we detected and rectified the outliers through data scaling and tenfold cross-validation using supervised methods. However, independent validation using an external dataset would still be warranted.

### Study Strengths and Limitations

The strength of this study includes a well-characterized and deeply phenotyped LVO patient cohort and the availability of radiological images, and infarct volume measures, which allowed the comparison of molecular markers with stroke severity and the outcomes. The discovery of actionable molecules implicated in the development of ischemic core and patient outcomes will be essential to better understanding AIS pathophysiology and improving treatment strategies. Machine learning approaches have been successfully used to integrate multi-omics data to predict hepatic carcinomas [62] and diabetes and classify stroke etiology [63]. This is the first human study reporting a joint evaluation using clinical, radiological, molecular, and artificial intelligence tools for predicting the functional outcomes of LVO strokes. The weaknesses include a relatively small sample size of LVO patients. We analyzed RNA sequencing data for miRNAs, which account for only 2% of the entire EV transcriptome. In addition, our study examined targeted metabolite profiles for 250 analytes using NMR. The use of global metabolomics (>3000 analytes) and their absolute quantifications would improve the detection of critical chemical features to predict stroke outcomes in large strokes. Even though the MISS patients were recruited from multiethnic populations, because of Oklahoma demographics, there is an over-representation of European Americans in this study, which limits the generalizability. Replication and validation of these results in other ethnic datasets would improve generalizability. 

## 5. Conclusions

In summary, no currently existing prediction models can guide stroke prognostication in LVOs with large core strokes. This study lays the groundwork for further research to incorporate new molecular characteristics into the current determinants, aiming to enhance their predictive accuracy. Ultimately, our work provides a framework for advancing stroke therapeutics and care by adding molecular markers to improve prognostication based on clinical assessments, radiological exams, and artificial intelligence to enhance patient care and functional outcomes. 

## Figures and Tables

**Table 1:** Clinical characteristics of study participants.

TRAIT		Large Infarct Volume (N = 43)	Small Infarct Volume (N = 42)	*p*-Value
Age (years)		64.9 ± 12.8	62.2 ± 16.4	0.40
BMI (kg/m^2^)		29.5 ± 7.0	29.6 ± 8.9	0.94
Glucose (mg/dL)		162.9 ± 96.7	128.5 ± 59.3	0.05
Gender/Male (%)		51	40	0.33
Ethnicity (%)	Caucasians	93	74	-
	African American	7	14	-
	Others	-	12	-
Systolic BP (mmHg)		158.1 ± 32.7	145.7 ± 21.9	0.04
Diastolic BP (mmHg)		83.7 ± 19.0	87.0 ± 15.1	0.40
Type 2 diabetes (%)		53	36	0.12
Smoking (%)		44	50	0.66
Stroke outcome		4.2 ± 1.9	2.6 ± 1.9	0.0004
NIHSS		12.6 ± 9.4	9.5 ± 8.0	0.11
Infarct Volume		117.4 ± 69.1	19.1 ± 14.1	7.43 × 10^−12^

Values are displayed as mean±SD; BMI: body mass index; BP: blood pressure; NIHSS: National Institute of Health Stroke Scale.

**Table 2:** Association of serum metabolites with infarct volume.

Metabolites	Biomarker Name	t.stat	*p*-Value	log2(FC)	Beta	SE	*p*-Value
Glucose	Glucose	2.24	2.84 ×10^−2^	0.89	0.51	0.17	3.23 ×10^−3^
S-HDL-FC%	Free cholesterol to total lipids ratio in small HDL	2.05	4.47 ×10^−2^	0.93	0.53	0.18	4.61 ×10^−3^
S-LDL-PL	Phospholipids in small LDL	2.67	9.68 ×10^−3^	0.98	0.34	0.19	7.74 ×10^−2^
Gln	Glutamine	2.16	3.43 ×10^−2^	1.97	0.63	0.37	9.14 ×10^−2^
Acetoacetate	Acetoacetate	2.09	4.09 ×10^−2^	1.18	0.36	0.16	2.97 ×10^−2^
GlycA	Glycoprotein acetyls	1.44	1.54 ×10^−1^	0.72	0.48	0.18	1.15 ×10^−2^
XL-HDL-P	Concentration of very large HDL particles	1.41	1.63 ×10^−1^	0.46	0.64	0.23	7.24 ×10^−3^
XL-HDL-CE	Cholesteryl esters in very large HDL	1.38	1.72 ×10^−1^	0.48	0.73	0.26	6.96 ×10^−3^
XL-HDL-C	Cholesterol in very large HDL	1.24	2.20 ×10^−1^	0.48	0.73	0.28	1.12 ×10^−2^
XL-HDL-L	Total lipids in very large HDL	1.10	2.77 ×10^−1^	0.44	0.69	0.29	1.97 ×10^−2^
XL-HDL-FC%	Free cholesterol to total lipids ratio in very large HDL	−1.29	2.02 ×10^−1^	−0.70	−0.75	0.29	1.21 ×10^−2^

SE: standard error; FC: fold change; data were analyzed using Metaboanalyst 5.0 and multivariate linear regression analysis adjusting for covariates of age, gender, and BMI.

## Data Availability

The data used and/or analyzed during the current study will be available from the corresponding author upon reasonable request.

## Supplementary Materials

The following supporting information can be downloaded at: https://www.mdpi.com/article/10.3390/jcm13195917/s1: Figure S1: Scatter plots depicting the correlation of selected serum metabolites associated with infarct volume; Figure S2: Heat maps depicting the correlation of significant miRNAs with serum metabolites; Table S1: Association of serum exosome miRNAs with stroke infarct volume; Table S2: Correlation of serum exosome miRNAs with NIHSS; Table S3: Correlation of serum exosome miRNAs with stroke outcome; Table S4: Correlation of serum metabolites with NIHSS; Table S5: Correlation of serum metabolites with stroke outcome; Table S6: Feature selection of metabolites for machine learning based on stroke outcome.
