# Supplementary material for: Improving Stroke Outcome Prediction Using Molecular and Machine Learning Approaches in Large Vessel Occlusion
Source: J Clin Med. 2024 Oct 3;13(19):5917. doi: 10.3390/jcm13195917 (PMC11477941; doi:10.3390/jcm13195917)
Supplement: Supplementary file 1 [file jcm-13-05917-s001.zip › jcm-3220246-supplementary.pdf]

Table S1: Association of serum exosome miRNAs with stroke infarct volume

miRNA	t.stat	P-value	log2(FC)	Beta	SE	P-Value	FDR
hsa-miR-144-5p	-4.68	1.09E-05	-4.55	-1.09	0.17	1.21E-08	2.62E-06
hsa-miR-486-5p	-3.65	4.62E-04	-1.44	-0.88	0.17	2.32E-06	2.50E-04
hsa-miR-101-3p	3.83	2.45E-04	0.53	0.85	0.17	2.95E-06	2.13E-04
hsa-miR-146b-5p	-3.26	1.59E-03	-2.32	-0.78	0.18	4.99E-05	2.15E-03
hsa-miR-501-3p	-2.47	1.55E-02	-1.54	-0.78	0.18	5.11E-05	1.84E-03
hsa-miR-21-5p	3.52	7.05E-04	0.70	0.72	0.17	8.77E-05	2.71E-03
hsa-miR-182-5p	-2.62	1.05E-02	-1.92	-0.76	0.18	9.15E-05	2.47E-03
hsa-miR-532-5p	-2.39	1.91E-02	-0.10	-0.69	0.18	2.08E-04	4.08E-03
hsa-miR-127-3p	-2.75	7.33E-03	-2.67	-0.71	0.18	2.33E-04	4.20E-03
hsa-miR-96-5p	-2.13	3.62E-02	-1.06	-0.70	0.18	2.57E-04	4.28E-03
hsa-miR-184	-2.32	2.28E-02	-2.30	-0.68	0.18	2.80E-04	4.04E-03
hsa-miR-409-3p	-2.81	6.25E-03	-2.05	-0.73	0.19	2.82E-04	3.80E-03
hsa-miR-625-3p	-3.31	1.36E-03	-2.03	-0.71	0.19	2.92E-04	3.72E-03
hsa-miR-140-3p	-3.45	8.77E-04	-1.54	-0.68	0.18	3.94E-04	4.73E-03
hsa-miR-125a-5p	-2.35	2.10E-02	-1.75	-0.65	0.18	5.01E-04	5.70E-03
hsa-miR-1307-3p	-2.45	1.64E-02	-0.61	-0.67	0.18	5.09E-04	5.50E-03
hsa-miR-99a-5p	-1.96	5.34E-02	0.46	-0.68	0.19	5.68E-04	5.84E-03
hsa-miR-1-3p	-2.26	2.68E-02	-0.76	-0.66	0.18	5.97E-04	5.86E-03
hsa-miR-130b-3p	-1.46	1.48E-01	-0.31	-0.64	0.18	7.83E-04	7.35E-03
hsa-miR-628-3p	-1.86	6.66E-02	-0.15	-0.59	0.18	1.44E-03	1.15E-02
hsa-miR-99b-5p	-2.46	1.59E-02	-0.28	-0.63	0.19	1.77E-03	1.32E-02
hsa-miR-142-3p	-2.69	8.66E-03	-1.88	-0.66	0.20	1.95E-03	1.36E-02
hsa-miR-139-3p	-2.54	1.30E-02	-1.78	-0.59	0.19	2.08E-03	1.41E-02
hsa-miR-574-3p	-2.58	1.18E-02	-2.26	-0.59	0.19	2.21E-03	1.45E-02
hsa-miR-485-3p	-1.91	6.00E-02	-2.17	-0.57	0.18	2.70E-03	1.71E-02
hsa-miR-181a-2-3p	-1.88	6.37E-02	0.06	-0.61	0.20	2.87E-03	1.77E-02

miRNA	t.stat	P-value	log2(FC)	Beta	SE	P-Value	FDR
hsa-miR-1180-3p	-2.97	3.93E-03	0.33	-0.57	0.19	2.93E-03	1.76E-02
hsa-miR-28-3p	-1.44	1.53E-01	-0.62	-0.56	0.19	3.53E-03	2.00E-02
hsa-let-7d-5p	-2.68	8.95E-03	-0.60	-0.53	0.19	5.36E-03	2.69E-02
hsa-miR-10a-3p	-2.29	2.45E-02	-0.70	-0.53	0.19	6.77E-03	3.25E-02
hsa-miR-660-5p	-3.27	1.59E-03	-0.97	-0.52	0.19	7.17E-03	3.29E-02
hsa-miR-324-5p	-1.87	6.52E-02	0.07	-0.51	0.19	7.32E-03	3.29E-02
hsa-miR-183-5p	-1.89	6.26E-02	0.02	-0.53	0.19	7.56E-03	3.27E-02
hsa-let-7f-5p	-2.12	3.71E-02	-1.26	-0.51	0.19	8.54E-03	3.30E-02
hsa-let-7e-5p	-1.64	1.04E-01	-0.83	-0.50	0.19	1.09E-02	3.78E-02
hsa-miR-16-5p	2.26	2.67E-02	0.18	0.46	0.19	1.65E-02	5.23E-02
hsa-miR-27a-3p	0.94	3.49E-01	0.19	0.45	0.19	1.68E-02	5.27E-02
hsa-miR-29c-3p	2.43	1.73E-02	0.74	0.43	0.18	2.05E-02	6.14E-02
hsa-miR-381-3p	-1.70	9.35E-02	0.18	-0.46	0.20	2.22E-02	6.55E-02
hsa-miR-103a-3p	-2.96	3.97E-03	-0.63	-0.45	0.20	2.58E-02	7.24E-02
hsa-miR-432-5p	-1.79	7.64E-02	-1.37	-0.39	0.19	4.31E-02	1.06E-01

SE: Standard error; FC: Fold change; FDR: False discovery rate; Data was analyzed using Metaboanalyst 5.0 and multivariate linear regression analysis adjusting for covariates of age, gender, and BMI

Table S2: Correlation of serum exosome miRNAs with NIHSS

miRNA	r	p-value
hsa-miR-28-3p	-0.527**	4.31E-07
hsa-miR-16-5p	0.515**	8.53E-07
hsa-miR-625-3p	-0.513**	9.78E-07
hsa-miR-142-3p	-0.504**	1.59E-06
hsa-miR-501-3p	-0.497**	2.36E-06
hsa-miR-1307-3p	-0.495**	2.68E-06
hsa-miR-485-3p	-0.486**	4.29E-06
hsa-miR-144-5p	-0.481**	5.42E-06
hsa-miR-96-5p	-0.460**	1.59E-05
hsa-miR-127-3p	-0.459**	1.61E-05
hsa-miR-409-3p	-0.455**	2.00E-05
hsa-miR-125a-5p	-0.444**	3.34E-05
hsa-miR-99b-5p	-0.423**	8.52E-05
hsa-miR-574-3p	-0.409**	1.48E-04
hsa-miR-486-5p	-0.405**	1.77E-04
hsa-miR-139-3p	-0.389**	3.29E-04
hsa-let-7e-5p	-0.383**	4.10E-04
hsa-miR-184	-0.371**	6.58E-04
hsa-miR-10a-3p	-0.368**	7.32E-04
hsa-miR-99a-5p	-0.360**	9.49E-04
hsa-let-7f-5p	-0.360**	9.56E-04
hsa-miR-182-5p	-0.350**	1.36E-03
hsa-miR-181a-2-3p	-0.342**	1.78E-03
hsa-miR-140-3p	-0.338**	2.00E-03
hsa-miR-101-3p	0.337**	2.09E-03
hsa-miR-660-5p	-0.336**	2.20E-03

miRNA	r	p-value
hsa-miR-1180-3p	-0.331**	2.51E-03
hsa-miR-146b-5p	-0.331**	2.54E-03
hsa-miR-628-3p	-0.323**	3.26E-03
hsa-miR-1-3p	-0.323**	3.28E-03
hsa-miR-324-5p	-0.320**	3.56E-03
hsa-miR-183-5p	-0.319**	3.75E-03
hsa-let-7d-5p	-0.304**	5.79E-03
hsa-miR-103a-3p	-0.302**	6.16E-03
hsa-miR-532-5p	-0.291**	8.51E-03
hsa-miR-130b-3p	-0.288**	9.21E-03
hsa-miR-381-3p	-0.246*	2.69E-02
hsa-miR-432-5p	-0.229*	3.97E-02

r: Pearson's correlation coefficient; NIHSS: National Institute of Health Stroke Scale

Table S3: Correlation of serum exosome miRNAs with stroke outcome

miRNA	r	p-value
hsa-miR-139-3p	-0.328**	8.67E-03
hsa-miR-140-3p	-0.322*	1.02E-02
hsa-miR-324-5p	-0.302*	1.60E-02
hsa-miR-127-3p	-0.296*	1.86E-02
hsa-miR-144-5p	-0.292*	2.03E-02
hsa-miR-625-3p	-0.291*	2.05E-02
hsa-miR-485-3p	-0.275*	2.94E-02
hsa-miR-501-3p	-0.266*	3.53E-02

r: Pearson's correlation coefficient

Table S4: Correlation of serum metabolites with NIHSS

Metabolites	Biomarker name	r	p-value
Acetoacetate	Acetoacetate	0.348**	5.21E-03
Glycerol	Glycerol	-0.307*	1.44E-02
S-LDL-C %	Cholesterol to total lipids ratio in small LDL	-0.302*	1.60E-02
XL-HDL-FC %	Free cholesterol to total lipids ratio in very large HDL	-0.293*	1.98E-02
S-HDL-FC %	Free cholesterol to total lipids ratio in small HDL	0.289*	2.18E-02
XL-HDL-CE	Cholesteryl esters in very large HDL	0.281*	2.57E-02
Albumin	Albumin	-0.268*	3.34E-02
XL-HDL-C	Cholesterol in very large HDL	0.261*	3.91E-02
XL-HDL-L	Total lipids in very large HDL	0.252*	4.61E-02
L-HDL-FC %	Free cholesterol to total lipids ratio in large HDL	0.252*	4.64E-02
XL-HDL-P	Concentration of very large HDL particles	0.251*	4.70E-02
M-LDL-TG %	Triglycerides to total lipids ratio in medium LDL	0.251*	4.76E-02
M-LDL-C %	Cholesterol to total lipids ratio in medium LDL	-0.249*	4.90E-02

r: Pearson's correlation coefficient; NIHSS: National Institute of Health Stroke Scale

Table S5: Correlation of serum metabolites with stroke outcome

Metabolites	Biomarker name	r	p-value
Acetoacetate	Acetoacetate	0.432**	1.22E-03
bOHbutyrate	3-Hydroxybutyrate	0.339*	1.31E-02
L-LDL-TG %	Triglycerides to total lipids ratio in large LDL	0.332*	1.52E-02
M-LDL-TG %	Triglycerides to total lipids ratio in medium LDL	0.318*	2.03E-02
L-HDL-TG %	Triglycerides to total lipids ratio in large HDL	-0.313*	2.23E-02
IDL-TG %	Triglycerides to total lipids ratio in IDL	0.306*	2.58E-02
XS-VLDL-TG %	Triglycerides to total lipids ratio in very small VLDL	0.298*	3.03E-02
L-HDL-CE	Cholesteryl esters in large HDL	0.291*	3.47E-02
L-HDL-C	Cholesterol in large HDL	0.286*	3.82E-02
L-HDL-PL	Phospholipids in large HDL	0.274*	4.69E-02
L-HDL-L	Total lipids in large HDL	0.273*	4.76E-02

r: Pearson's correlation coefficient

Table S6: Feature selection of metabolites for machine learning based on stroke outcome

Metabolites	Biomarker name	F-regression		Chi-square test	
		F	P-value	X^2	P-value
Glucose	Glucose	7.79	6.58E-03	9.36	2.21E-03
Total-FA	Total fatty acids	2.61	1.10E-01	6.63	1.00E-02
XL-VLDL-TG %	Triglycerides to total lipids ratio in very large VLDL	2.34	1.30E-01	8.85	2.94E-03
XXL-VLDL-CE %	Cholesteryl esters to total lipids ratio in chylomicrons and extremely large VLDL	1.76	1.88E-01	14.32	1.54E-04
XXL-VLDL-C %	Cholesterol to total lipids ratio in chylomicrons and extremely large VLDL	1.75	1.90E-01	13.79	2.05E-04
L-VLDL-TG %	Triglycerides to total lipids ratio in large VLDL	1.64	2.04E-01	4.42	3.54E-02
Creatinine	Creatinine	1.24	2.68E-01	32.84	1.00E-08
XXL-VLDL-TG %	Triglycerides to total lipids ratio in chylomicrons and extremely large VLDL	0.92	3.41E-01	6.66	9.86E-03
LA %	Ratio of linoleic acid to total fatty acids	0.90	3.46E-01	3.85	4.98E-02

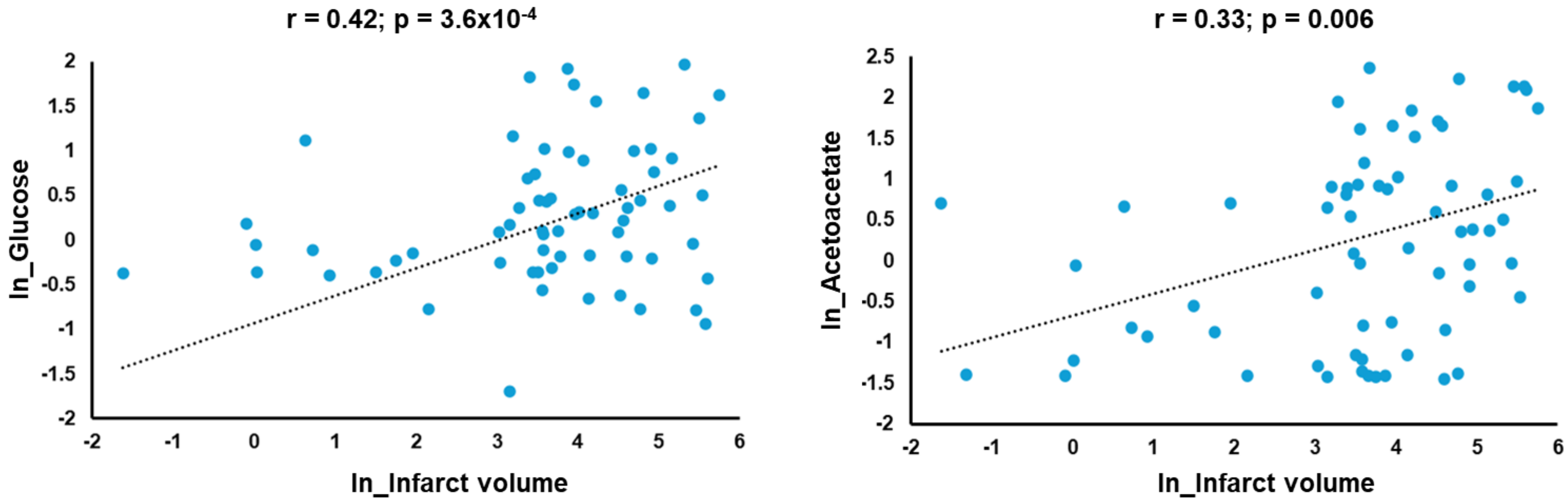

Figure S1: Scatter plots depicting the correlation of selected serum metabolites associated with infarct volume

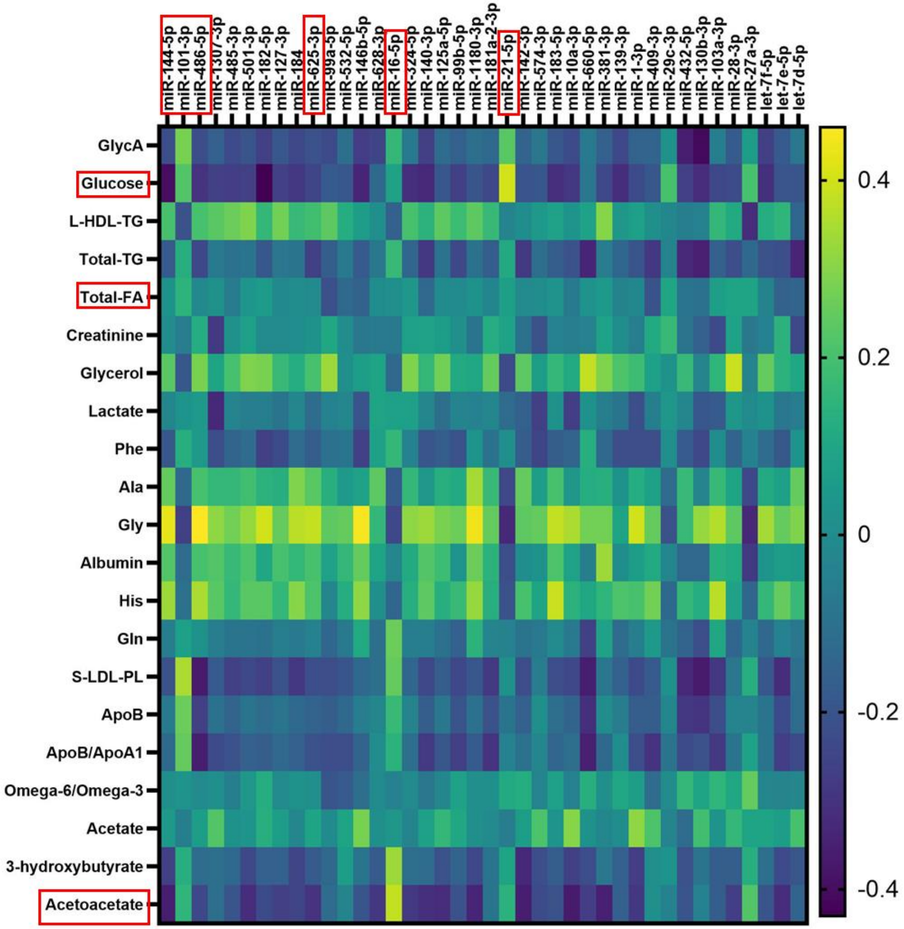

Figure S2: Heat maps depicting the correlation of significant miRNAs with serum metabolites

GlycA: Glycoprotein acetyls; L-HDL-TG: Triglycerides in large HDL; Total-TG: Total triglycerides; Total-FA: Total Fatty acids; Phe: Phenylalanine; Ala: Alanine; Gly: Glycine; His: Histidine; Gln: Glutamine; S-LDL-PL: Phospholipids in small LDL; ApoB: Apolipoprotein B; ApoB/ApoA1: Ratio of apolipoprotein B to apolipoprotein A1
